# Prosocial behavior according to sex in school adolescents immersed in violent contexts in the department of Córdoba, Colombia

**DOI:** 10.1192/j.eurpsy.2022.477

**Published:** 2022-09-01

**Authors:** E.P. Ruiz Gonzalez, A.M. Romero Otalvaro, J. Velez Carvajal, K. Seña Giraldo, M. Tuiran Catalan

**Affiliations:** Universidad Pontificia Bolivariana, Cordoba, Monteria, Colombia

**Keywords:** violent contexts, Prosocial behavior, Adolescents

## Abstract

**Introduction:**

Four specific forms of violence have been identified in the socialization process of children, and these are: “violent discipline and exposure to domestic abuse; violence at school; violent deaths among adolescents; and sexual violence ”. (UNICEF, 2017, p2), In this regard Redondo & Inglés (2014) affirm that it is increasingly evident the need to promote prosocial behavior models based on empathy and assertiveness in educational institutions, in order to avoid the appearance of violent demonstrations.

**Objectives:**

Analyze the levels of prosocial behavior according to sex in adolescents

**Methods:**

A descriptive, cross-sectional study was conducted in 105 (N = 105) adolescents. A sociodemographic survey was used to investigate aspects related to the study objective and the Prosocial Behavior questionnaire by Martorell and Gonzales (1922) to measure prosocial behavior.

**Results:**

57.7% of the adolescents evaluated presented adequate prosocial behaviors. When examining the difference between sex, the expected values were initially verified, which indicated the feasibility of performing a student’s T; As can be seen in Table 1, the mean corresponding to the female sex was 49.62%, in contrast to a mean of 49.93% for the male sex, indicating the absence of statistically significant differences.

**Figure:**
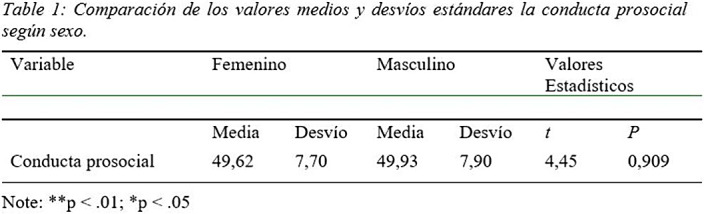

**Conclusions:**

It was concluded that the higher the optimal levels of empathy, the lower the aggressive behavior presented by teenagers.

**Disclosure:**

No significant relationships.

